# Chromosome transmission in BC_4_ progenies of intergeneric hybrids between *Saccharum* spp. and *Erianthus arundinaceus* (Retz.) Jeswiet

**DOI:** 10.1038/s41598-019-38710-8

**Published:** 2019-02-21

**Authors:** Shan Yang, Kai Zeng, Ke Chen, Jiayun Wu, Qinnan Wang, Xueting Li, Zuhu Deng, Yongji Huang, Fei Huang, Rukai Chen, Muqing Zhang

**Affiliations:** 1 National Engineering Research Center for Sugarcane, Fujian Agriculture and Forestry University, Fuzhou, 350002 China; 2 Key Lab of Sugarcane Biology and Genetic Breeding, Ministry of Agriculture, Fujian Agriculture and Forestry University, Fuzhou, 350002 China; 3 Guangdong Provincial Bioengineering Institute, Guangzhou Sugarcane Industry Research Institute, Guangzhou, 510316 China; 4 State Key Laboratory for Conservation and Utilization of Subtropical Agro-bioresources, Guangxi University, Nanning, 530004 China

## Abstract

Intergeneric hybrids between *Saccharum* spp. and *Erianthus arundinaceus* and clones derived from these hybrids and backcrosses to *Saccharum* spp. were used to study the transmission of *E*. *arundinaceus* chromosomes by genomic *in situ* hybridization (GISH). True hybrid progenies were precisely identified using PCR with a primer pair, AGRP52/53. The results showed that AGRP52/53 was an *E*. *arundinaceus*-specific primer pair and could be used as molecular marker to assist breeding. EaHN92, a 364 bp *E*. *arundinaceus*-specific tandem repeat satellite DNA sequence, was cloned from the *E*. *arundinaceus* clone HN92–105 with AGRP52/53, and was localized on sub-telomeric regions of all *E*. *arundinaceus* chromosomes. YCE06–61, a BC_3_ progeny, had 7 *E*. *arundinaceus* chromosomes and its progenies had approximately 1–6 *E*. *arundinaceus* chromosomes. The number of *E*. *arundinaceus* chromosomes in true hybrids appeared as Gaussian distribution in 3 cross combinations. In addition, GISH detected intergeneric chromosome translocation in a few progenies. Hence, screening clones containing approximately 1–2 *E*. *arundinaceus* chromosomes without translocation could be used for sorting and sequencing *E*. *arundinaceus* chromosomes. This study provides a method for breeders to select true hybrid progenies between *Saccharum* spp. and *E*. *arundinaceus*, which will accelerate this intergeneric hybridization breeding.

## Introduction

Sugarcane (*Saccharum* spp.) belongs to the genus *Saccharum*, family *Gramineae*, and is an important energy crop. *Saccharum* consists of six species, namely *S*. *officinarum*, *S*. *sinense*, *S*. *bareri*, *S*. *edule*, *S*. *robustum*, and *S*. *spontaneum*. *Saccharum* has a close genetic relationship with *Miscanthus*, *Sclerostachya*, *Erianthus*, and *Narenga*, which constitute an interbreeding group called the “*Saccharum* complex”^1,2^. In 1996, D’Hont *et al*.^3^ reported that modern sugarcane cultivars possess approximately 120 chromosomes, with 70–80% derived from *S*. *officinarum*, 10–20% derived from *S*. *spontaneum*, and a few chromosomes derived from interspecific recombination.

Commercial sugarcane cultivars are derived from interspecific hybridization of different *Saccharum* species. Sugarcane has low heterogeneity and a narrow genetic base, which limits its yield, quality, and resistance^4^. However, another genus, *Erianthus*, if introduced into sugarcane, could overcome these limitations. *E*. *arundinaceus* has many desirable agronomic traits for sugarcane breeding, such as broad adaptability, disease resistance, drought resistance, and a high biomass^5^. The intergeneric F_1_ progeny between *Saccharum* spp. and *E*. *arundinaceus* cannot be developed using the “nobilization” hybridization strategy to increase cane yield and restore high sugar content because pollen from the hybrid clones are sterile^6^. In 2001, hybridization between an F_1_ clone as the female parent and a sugarcane cultivar as the male parent at the Hainan sugarcane breeding station in China was achieved and produced intergeneric BC_1_ progenies. BC_2_ progenies were produced in two years later. Meanwhile, sugarcane breeders found that the pollen fertility of the BC_2_ progenies was recovered^7^. Thus, a new hybridization strategy using a sugarcane cultivar as the female parent and a BC_2_ clone as the male parent has since been used^8^. Based on this strategy, our group also successfully created intergeneric BC_3_ and BC_4_ progeny.

Repetitive DNA sequences represent a large part of the eukaryotic genome, have different number of copies in genome and possess various specific features^9^. Consequently, repetitive sequences are sources of molecular markers that are useful in plant genetic studies, and have been cloned from many higher plant genera for use in phylogenetic studies or introgression breeding programs^10–13^. They diverged rapidly during evolution and are constantly homogenized, giving rise to sequences that are species-specific, genus-specific, and even chromosome-specific^14,15^. Moreover, their localization by situ hybridization provide important information on chromosome structure^16^. Accordingly, these repetitive DNA sequences have provided new molecular tools to investigating genetic diversity and phylogenetic relationships in the *Saccharum* complex and improving the efficiency of modern molecular breeding of sugarcane.

The genomic *in situ* hybridization (GISH) technique is used to study chromosomal structure, exchange, and mode of transmission and inheritance between parent and filial generations^17^. The mode of chromosome inheritance has been studied through the processes of nobilization breeding between *S*. *officinarum* and wild germplasm^18^. This work has indicated that chromosome inheritance occurs through n + n, 2n + n, n + 2n, and 2n + 2n transmission^19–22^. Wu *et al*.^23^ studied the chromosomal inheritance of hybrid progeny generated between *Saccharum* spp. and *E*. *arundinaceus* and found that the mode of chromosome inheritance was n + n transmission in the hybrid F_1_ progenies, 2n + n transmission in 9 of 13 hybrid BC_1_ progenies, and more than 2n + n transmission in the 4 of 13 hybrid BC_1_ progenies. However, previous studies have not reported the pattern of *E*. *arundinaceus* chromosome transmission from parents to progeny in the BC_4_ generation.

Distant hybrid utilization of *E*. *arundinaceus* in sugarcane has made great progress in recent years. Unfortunately, the genomics of *Saccharum* spp. and *E*. *arundinaceus* are far behind those of cereal crops since *Saccharum* spp. and *E*. *arundinaceus* are polyploid plants with large genomes and many homologous sequences^24,25^. However, researchers can use the genome sequencing strategy for wheat, which is called “break up the whole into parts,” or “BAC BY BAC,” for *Saccharum* spp. or *E*. *arundinaceus*. In this strategy, the wheat genome is broken down into a single chromosome or a single chromosome arm, which can be used to build the BAC library and physical map^26^. Therefore, sorting and sequencing *E*. *arundinaceus* chromosomes can be achieved by screening BC_4_ generation clones that contain 1 or 2 *E*. *arundinaceus* chromosomes.

Here, we aimed to determine whether EaHN92, the PCR product of AGRP52/53 as a primer, is an *E*. *arundinaceus*-specific tandem sequence, and whether it can hybridize with every *E*. *arundinaceus* chromosome through FISH. We also selected 3 cross combinations of intergeneric hybrid BC_4_ (1511, 1514, and 1625) as research materials to identify true hybrids in a BC_4_ population using PCR with an AGRP52/53 primer pair. We then used GISH to clarify the pattern of *E*. *arundinaceus* chromosome transmission to determine the presence of intergeneric chromosomal translocation and to screen clones that contained approximately 1 or 2 *E*. *arundinaceus* chromosomes to sort and sequence the *E*. *arundinaceus* chromosomes. This work will provide a basis for subsequent genome research in *E*. *arundinaceus* and *Saccharum* spp.

## Materials and Methods

### Plant materials

A total of 74 clones from three intergeneric BC_4_ populations, named 1511, 1514, and 1625 respectively, were selected in this study. These clones were from a crossing combination between a sugarcane cultivar (♀) and the clone YCE06–61 (♂). The latter was a clone in the BC_3_ generation derived from *E*. *arundinaceus* containing 7 *E*. *arundinaceus* chromosomes^8^. There were 20 clones from population 1511, 28 clones from population 1514 and 26 clones from population 1625 (Table 1). A further 17 clones from *Saccharum*, *E*. *arundinaceus* and progenies of *E*. *arundinaceus* were also included (Table 2).

**Table 1:** BC_4_ progenies between *Saccharum* spp. and *E*. *arundinaceus*.

Progenies	Generations	Female parent	Male parent	Number of progenies
YCE06–61	BC_3_	ROC10	YCE03-01 (BC_2_)	
1511	BC_4_	FN02-3924	YCE06–61 (BC_3_)	20
1514	BC_4_	FN02-6427	YCE06–61	28
1625	BC_4_	HoCP01-564	YCE06–61	26

**Table 2:** *Saccharum*, *Erianthus* and the hybrids used in the study.

No.	Genera/Species	Materials	Origin	Chromosome No.
1	*S*. *officinarum*	Badila	Indonesia	80
2	*S*. *officinarum*	Fruit sugarcane	China	80
3	*S*. *officinarum*	Black Cheribon	Indonesia	80
4	*S*. *robustum*	51NG63	New Guinea	80
5	*S*. *spontaneum*	FJ89-1-21	Fujian, China	96
6	*S*. *spontaneum*	Laos-2	Laos	Unknown
7	*S*. *spontaneum*	Thailand-1	Thailand	Unknown
8	*S*. *sinense*	GX-*S*. *sinense*	Guangxi, China	Unknown
9	*S*. *sinense*	GD-*S*. *sinense*	Guangdong, China	Unknown
10	*S*. *sinense*	Uba	China	Unknown
11	*S*. *bareri*	Mungo	India	Unknown
12	*S*. *bareri*	Katha	India	Unknown
13	*E*. *arundinaceus*	HN92–105	Hainan, China	60
14	*S*. *officinarum* × *E*. *arundinaceus* (F_1_)	YCE96-40	Hainan, China	69
15	F_1_ × Sugarcane cultivar	YCE01–102 (BC_1_)	Hainan, China	118
16	BC_1_ × Sugarcane cultivar	YCE03–01 (BC_2_)	Hainan, China	119
17	Sugarcane cultivar × BC_2_	YCE06–61 (BC_3_)	Hainan, China	114

### Genomic DNA extraction

Young leaves of different individual plants were cut, ground with liquid nitrogen and genomic DNA was extracted using the traditional CTAB method following the method of Mace *et al*.^27^.

### Polymerase chain reaction (PCR)

PCR identification of true hybrid progeny was conducted. A PCR reaction mixture was prepared on ice (Table 3) and carried out in a thermal cycler (ABI, USA) using the primer pairs AGRP52 and AGRP53 (AGRP52: 5′-AGGAAGTTATGGTGGAGTAT-3′; AGRP53: 5′-CGCCATTCCTATTGC-3′) following the method of Alix *et al*.^28^. The PCR program was performed as follows: pre-denaturation at 95 °C for 3 min, 30 cycles of 95 °C for 20 s, 55 °C for 20 s, 72 °C for 5 s, and 72 °C for 3 min. PCR products were tested using 1.5% agarose gel electrophoresis.

**Table 3:** PCR reaction mixture.

Components	Add volume (μL)
ddH_2_O	15
10 × Ex buffer (Mg^2+^ plus)	2
dNTP (2.5 mM each)	1.6
AGRP52 (10 μM)	0.5
AGRP53 (10 μM)	0.5
Template (gDNA) (50 ng/μL)	0.3
Ex Taq (5 U/μL)	0.1
Total volume	20

Using HN92–105 as a template, positive PCR products were purified via using the OMEGA EZNA Gel Extraction Kit (Omega, USA). The purified products were stored at −20 °C, as standby, and designated EaHN92. EaHN92 was then cloned into a pMD19-T vector (Takara, Japan) and transformed into an *E*. *coli* DH5α competent cell (Takara, Japan). The recombinant clones were grown in LB culture medium with ampicillin (100 μg/mL). Five clones were randomly selected for sequencing by the Sangon Biotech Company (Shanghai, China).

### Chromosome preparation and slide preparation

Plants of the clone HN92–105 were grown in a pot with sandy loam soil. After growing for 60 days in summer root tips were cut at 9:00am every three days. Cane stalks of YCE06–61, and clones from populations 1511 (19 clones), 1514 (27 clones) and 1625 (24 clones) were cut into single eye setts, planted into trays which were kept at 25°C and covered by gauze to keep moist. After 5 days when the length of the roots were about 2–3 cm root tips were cut at 9:00 am. The root tips were pretreated with saturated dichlorobenzene solution at room temperature for 2.0 h to accumulate metaphase cells, placed into a fixation solution with a ratio of ethanol to acetic acid of 3:1 (v/v) for 18 h, and then stored at −20 °C in 75% ethanol solution until use. Chromosomal slide preparation was the same for FISH and GISH. The fixed roots were washed in water and digested at 37 °C for 150 min in an enzyme solution containing 8% Onozuka R10 cellulose (Yakult, Tokyo, Japan), 2% pectinase (Sigma, USA) and 1% pectolyase Y-23 (Yakult, Tokyo, Japan). The meristematic cells of root tips were squashed on a clean slide in a drop of fixation solution, then air-dried and stored at −20 °C until use.

### Fluorescence *in situ* hybridization (FISH) and genomic *in situ* hybridization (GISH) procedures

EaHN92 sequence was labeled with Biotin-16-dUTP (Roche) as the FISH probe, carried out using the procedures of PCR identification of true hybrid progeny. FISH experiments were performed according to Panwar *et al*.^29^ with some modifications. The 50 μL hybridization mixture containing 5 μL of the labeled FISH probe, 25 μL deionized formamide, 5 μL dextran sulfate, 10 μL 20 × SSC and 5 μL ddH_2_O was denatured at 97 °C for 10 min, and then placed immediately in ice-water for 10 min. Each chromosomal slide was denatured at 80 °C for 3 min in the denaturation solution containing 70% deionized formamide and 2 × SSC, dehydrated in a series of precool ethanol solutions (75%, 95%, and 100% ethanol), and incubated in a humid box with 2 × SSC at 37 °C for 20 h. Post-hybridization washes were performed sequentially, once in 2 × SSC at 42 °C for 5 min, twice in 20% deionized formamide and 2 × SSC at 42 °C for 5 min, twice in 2 × SSC at 42 °C for 5 min, once in 2 × SSC at room temperature for 5 min, and once in 4 × SSC and 0.2% Tween-20 for 5 min at room temperature.

Genomic DNA from HN92–105 (*E*. *arundinaceus*) was labeled with Biotin-16-dUTP (Roche) as probes for GISH. Genomic DNA from Badila (*S*. *officinarum*) were not labeled with a biomarker as the blocking agent in GISH. GISH was performed in accordance with D’Hont *et al*.^30^ with some modifications. The 50 μL hybridization mixture containing 2 μL of the 100 ng/μL labeled genomic probe of HN92–105, 3 μL of the 100 ng/μL unlabeled genomic probe of Badila, 25 μL deionized formamide, 5 μL dextran sulfate, 10 μL 20 × SSC and 5 μL ddH_2_O was denatured at 97 °C for 10 min. The following procedures were the same as with FISH except for post-hybridization washing, which was twice in 20% deionized formamide and 2 × SSC at 42 °C for 8 min.

The Biotin-labeled probe was detected by Avidin D, Rhodamine 600 (XRITC), and a Biotinylated anti-avidin antibody (Vector Laboratories, Burlingame, CA). Finally, chromosomes were counterstained with 30 μL/slide Vectashield antifade solution (Vector Laboratories, concentration of 10 μg/mL DAPI) and mounted with a coverslip. FISH and GISH signals were captured using an AxioScope A1 Imager fluorescent microscope (Carl Zeiss, Gottingen, Germany), of which the blue and red fluorescence signal were excited in DAPI and Texas-red channel respectively. Images were processed using AxioCam MRc5 and AxioVision v.4.7 software (Carl Zeiss, Gottingen, Germany). For each sample, the number of *E*. *arundinaceus* chromosomes was calculated as a range from observations of 10 to 15 cells in metaphase.

## Results

### Analysis of EaHN92 repeated sequence

The repeat units of EaHN92 tandem repeat sequences was 364 bp satellite DNA sequence, which was cloned from *E*. *arundinaceus* HN92–105. The EaHN92 specific repeated sequence was submitted to NCBI database (Accession number: MH133205). Its homology was estimated using a nucleotide blast tool in the NCBI database, which showed 93% homology with EaCIR1 cloned from *E*. *arundinaceus* (Accession number: Y13453.1), 81% homology with SSCIR2 cloned from *S*. *spontaneum* (Accession number: Y13452.1) and 79% homology with SOCIR1 cloned from *S*. *officinarum* (Accession number: Y13451.1).

### Analysis of fluorescence FISH results

**Figure 1:**
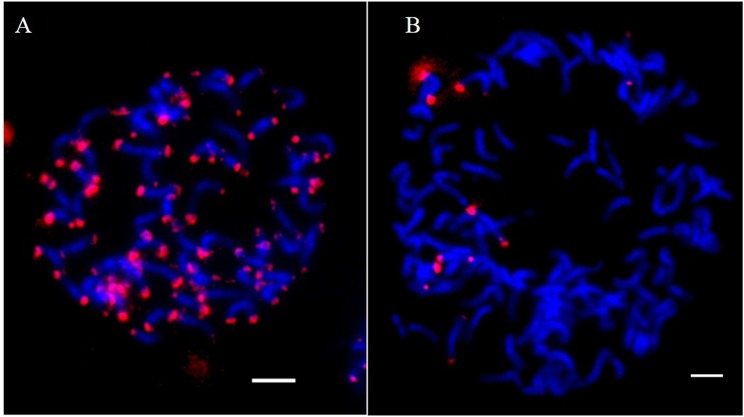
FISH analysis of EaHN92 labeled on mitotic chromosomes. (**A**) HN92–105 (2n = 6x = 60); (**B**) YCE06–61 contained 7 *E*. *arundinaceus* chromosomes. Red represents hybridization sites of EaHN92. All chromosomes are shown in blue. Scale bars: 5 μm.

**Figure 2:**
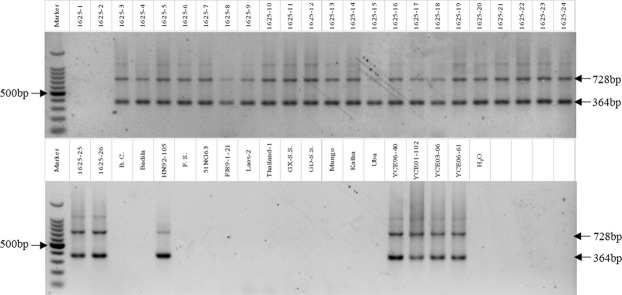
Electrophoretogram of 1625 progenies and sugarcane germplasm. Marker: 100 bp DNA ladder.

The FISH experiment using the repeat sequence EaHN92 as a probe identified EaHN92 hybridization sites in sub-telomeric regions at one or both ends of 60 chromosomes in HN92–105 (Fig. 1A). EaHN92 hybridization sites were also detected in sub-telomeric regions at both ends of 7 chromosomes in YCE06–61 (Fig. 1B). These results indicated that EaHN92 was a 364 bp *E*. *arundinaceus*-specific sequence and that AGRP52/53 was an *E*. *arundinaceus* specific primer pair, which could be used through PCR to identify true hybrid progeny, generated between *Saccharum* spp. and *E*. *arundinaceus* (Figs 1B, 2).

### PCR identification of true hybrids between *Saccharum* spp. and *E*. *arundinaceus*

**Figure 3:**
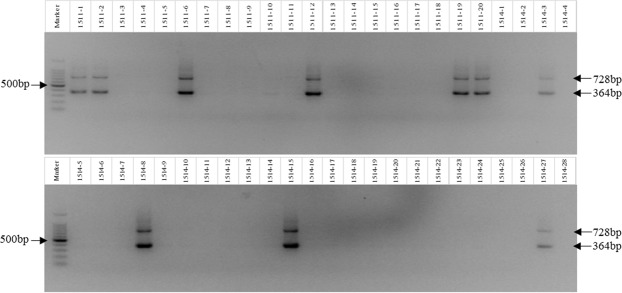
Electrophoretogram of 1511 and 1514 progenies. Marker: 100 bp DNA ladder.

A preliminarily evaluation of hybrid progeny of BC_4_ crosses was conducted using PCR and detected two electrophoretic bands (364 bp, 728 bp, respectively) (Figs 2, 3). These results indicated that the two electrophoretic bands clones without *E*. *arundinaceus* chromosomes were not amplified. The true hybrid rate of the BC_4_ populations 1511, 1514, and 1625 were 30.0%, 14.3%, and 92.3%, respectively (Figs 2, 3; Supplementary information dataset 1).

### Analysis of GISH results

**Figure 4:**
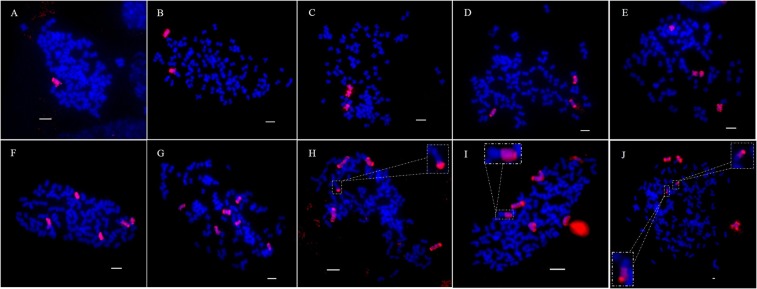
GISH analysis of BC_4_ progenies between *Saccharum* spp. and *E*. *arundinaceus*. *E*. *arundinaceus* chromosomes are shown in red and *Saccharum* spp. chromosomes are shown in blue. (**A**) 1625-13: 1 *E*.; (**B**) 1625-10: 2 *E*.; (**C**) 1625-19: 3 *E*.; (**D**) 1625-25: 3 *E*.; (**E**) 1625-23: 4 *E*.; (**F**) 1625-26: 5 *E*.; (**G**) 1514-8: 6 *E*.; (**H**) 1625-4: 4 *E*. + 1 *E*./*S*.; (**I**) 1625-7: 4 *E*. + 1 *E*./*S*.; (**J**) 1625-22: 4 *E*. + 2 *E*./*S*. Arrowheads in (**H**) (**I**) and (**J**) show translocated chromosomes. *E*. and *S*. indicate *E*. *arundinaceu*s chromosomes and *Saccharum* spp. chromosomes, respectively. *E*./*S*. indicates translocation of the *E*. *arundinaceu*s chromosome and *Saccharum* spp. chromosome. Scale bars: 5 μm.

The results of GISH detecting experiment indicated that the clones which were identified as being true hybrids via PCR identification contained 2, 4, and 6 *E*. *arundinaceus* chromosomes in the case of population 1511, 4 and 6 *E*. *arundinaceus* chromosomes in the case of population 1514, and approximately 1–6 *E*. *arundinaceus* chromosomes in the case of population 1625 (Table 4). The percentage of approximately 1–6 *E*. *arundinaceus* chromosomes of true hybrids appeared as Gaussian distribution in the 3 populations, of which 4 *E*. *arundinaceus* chromosomes (36.67%) made up the largest proportion, followed by 3 *E*. *arundinaceus* chromosomes (23.33%), 5 *E*. *arundinaceus* chromosomes (16.67%), 2 and 6 *E*. *arundinaceus* chromosomes (10.00%) and 1 *E*. *arundinaceus* chromosome (3.33%) (Table 4). These results revealed that the number of *E*. *arundinaceus* chromosome in transmission of BC_3_ to BC_4_ progenies were approximately reduced by half, but there would were special cases in this transmission where reduction was more or less than half (Fig. 4; Table 4; Supplementary information dataset 2). Intergeneric chromosomal translocation occurred between *Saccharum* spp. and *E*. *arundinaceus* in the clones 1625–4, 1625–7, and 1625-22; only one chromosome translocated in 1625-4 and 1625-7 and two chromosomes translocated in 1625-22 (Fig. 4H–J). The chromosomal translocation in 1625-4 occurred in the terminal regions (Fig. 4H). The chromosomal translocations in 1625-7 and 1625-22 occurred in the centromeric regions (Fig. 4I, J).

**Table 4:** Chromosome transmission in BC_4_ progenies of *Saccharum* spp. × *E*. *arundinaceus*.

Observed number of *E*.*arundinaceus* chromosomes	No. of clones	Total	Percentage
1	1625-13	1	3.33%
2	1511-1, 1625-10, 1625-21	3	10.00%
3	1625-6, 1625-8, 1625-12, 1625-14, 1625-18, 1625-19, 1625-25	7	23.33%
4	1511–12, 1511–19, 1511–20, 1514–15, 1514–25, 1625-3, 1625-9, 1625-17, 1625-23, 1625-24, 1625-20	11	36.67%
5	1625-4, 1625-5, 1625-7, 1625-11, 1625–26	5	16.67%
6	1511-6, 1514-8, 1625-22	3	10.00%

Note: 1511-2, 1514-3, 1625-15, and 1625-16 were non-survival.

## Discussion

In eukaryotes, a significant fraction of the genome is comprised of repetitive DNA sequences and this component is often greater than the coding sequence component^31^. Researchers have shown that the repetitive DNA sequences play an important role in numerous cell processes and species evolution^32^. Consequently, understanding the contents and origins of repetitive DNA sequences represents an important step towards completely deciphering the organization and function of the genome sequence^33^. Satellite DNA sequence is a type of repetitive DNA sequence used as molecular marker to assist breeding. In 1998, Alix *et al*.^28^ developed an *E*. *arundinaceus*-specific primers pair, AGRP52/53, based on the *E*. *arundinaceus*-specific satellite DNA sequence EaCIR1. However, earlier workers believed that the AGRP52/53 cannot be used as molecular marker for identifying true intergeneric hybrids of *Saccharum* spp. and *E*. *arundinaceus* because two of the *E*. *arundinaceus* chromosomes could not hybridize with EaCIR1 in the FISH experiment. Our FISH results were different from that of Alix *et al*.^28^ in indicating that the 364 bp sequence EaHN92 is actually an *E*. *arundinaceus*-specific sequence and hybridized with all *E*. *arundinaceus* chromosomes in the subtelomeric regions. Therefore, the EaHN92 sequence is an *E*. *arundinaceus*-specific tandem repeat sequence.

Intergeneric hybrid populations between *S*. *officinarum* and *E*. *arundinaceus* often turn out to be of false hybrids due to the high selfing rate^34^. Therefore, in 2002, Deng *et al*.^35^ used isozyme markers to identify true hybrid progeny generated between *E*. *arundinaceus* and *Saccharum* spp. However they could not identify the true hybrids because there were many similar bands in different parents. Following this work, SSR and 5 S rDNA markers were used to identify the true hybrid progeny^36,37^. In 2004, Zheng *et al*.^38^ identified the true hybrid progeny of *E*. *arundinaceus* through PCR using the primers, EF1/ER1 and EF2/ER2, which were based on the internal transcribed spacer (ITS) sequence of *E*. *arundinaceus*. However, in 2010, Deng *et al*.^22^ found that it was necessary to combine the results of PCR using EF1/ER1 or EF2/ER2 as primers to identify the true hybrid BC_2_ progenies generated from *E*. *arundinaceus* and *Saccharum* spp. In our study, true intergeneric hybrids between *Saccharum* spp. and *E*. *arundinaceus* could be rapidly and precisely identified using PCR with an AGRP52/53 primer pair. This approach could have useful wide application.

In recent years, much research has been carried out on chromosome transmission of different generations derived from hybrids between *Saccharum* spp. and *E*. *arundinaceus*. Wu *et al*.^23^ reported the mode of chromosome transmission was “n + n” in the F_1_ generation between *E*. *arundinaceus* and *S*. *officinarum* and the mode of chromosome transmission was “2n + n” in most BC_1_ generations produced between F_1_ and sugarcane cultivars. However, in some cases the chromosomes transmitted were more than “2n + n” in the BC_1_ generation. Huang *et al*.^8^ and Piperidis *et al*.^39,40^ reported that the mode of chromosome transmission was “n + n” in the BC_2_ and BC_3_ generation. In fact, previous studies reported the number of *E*. *arundinaceus* chromosomes was approximately 28–29, 22–31, 8–17 and 4–8 in the F_1_, BC_1_, BC_2_ and BC_3_ generation respectively^8,23^. In this study, the number of *E*. *arundinaceus* chromosomes was approximately 1–6 in the BC_4_ populations of *Saccharum* spp. and YCE06–61 with most having 3–4 *E*. *arundinaceus* chromosomes. YCE06–61 contains 7 *E*. *arundinaceus* chromosomes, and therefore the number of *E*. *arundinaceus* chromosomes of true BC_4_ hybrids appeared as Gaussian distribution in 3 cross combinations. In distant hybridization of plants, the complete and partial elimination of chromosome from one parent has been observed in crosses covering Gramineae and other species^41^. Such elimination appeared to be a common and nonradom event. Based on observation on meiosis behavior of pollen mother cells, Lin *et al*.^42^ found that chromosomes unevenly separated to new cells during meiosis in the F_1_ generation of *S*. *officinarum* and *Erianthus rockii* because *E*. *rockii* chromosomes lagged and lost. Unfortunately, the exact mechanism of this phenomenon is still not clear and need to be investigated further.

Translocated chromosomes are stable sources for transmitting hereditary information to the progeny in plant distant hybridization. In this study, we found intergeneric translocated chromosomes between *Saccharum* and *E*. *arundinaceus* in three clones of the BC_4_ generation, of which one occurred in the terminal regions and two occurred in the centromeric regions. Earlier studies had not reported intergeneric translocated chromosome in YCE06–61^8^. However, an intergeneric chromosomal translocations were reported within the BC_1_, BC_2_ and BC_3_ generation generated between *Saccharum* spp. and *E*. *arundinaceus*^8,23,39^. This indicates that intergeneric translocated chromosomes can arise randomly in different generations.

In this study, we identified clones in the BC_4_ generation with 1 or 2 *E*. *arundinaceus* chromosomes that did not appear to be translocated for sorting and sequencing of the *E*. *arundinaceus* chromosomes. Researchers around the world have pursued *S*. *spontaneum* and *S*. *officinarum* genome sequencing but have not achieved success due to the presence of many homologous sequences due to high level polyploidy and very complex sequence data. Garsmeur *et al*.^43^ achieved a mosaic monoploid reference sequence for sugarcane from R570 BAC clones, but missed many sequences because the BAC clones mostly distributed in sorghum gene-rich distal chromosomal regions. In 2018, Zhang *et al*.^44^ had achieved allele-defined genome of the autopolyploid *S*. *spontaneum* L. successfully, which it was an important finding for sugarcane. However, this genome map of *S*. *spontaneum* L. missed telomere sequences, many homologous sequences and repeated sequences. Therefore, in order to achieve a high-accuracy genome sequence of *E*. *arundinaceus*, we suggest that those performing *E*. *arundinaceus* genome research use the “BAC BY BAC” sequencing strategy since *E*. *arundinaceus* is a hexaploid plant with a large and complex genome. Although genome research of *Saccharum* and its relative genus polyploid plants is very difficult, this study provides new techniques to aid researchers.

## Conclusion

True hybrid progenies could be rapidly and precisely identified using PCR with an *E*. *arundinaceus*-specific primer pair, AGRP52/53. EaHN92, a 364 bp *E*. *arundinaceus*-specific sequence, was cloned from HN92–105 with AGRP52/53 and was localized on sub-telomeric regions of all *E*. *arundinaceus* chromosomes using FISH. According to the results of GISH, the number of *E*. *arundinaceus* chromosome in BC_4_ population ranged from 1 to 6 and appeared as Gaussian distribution. Identifying true hybrid clones with approximately 1–2 *E*. *arundinaceus* chromosomes without translocation using GISH could be used for sorting and sequencing *E*. *arundinaceus* chromosomes.

## Supplementary information

Dataset 1

Dataset 2
