# Supplementary material for: Chromosome transmission in BC4 progenies of intergeneric hybrids between Saccharum spp. and Erianthus arundinaceus (Retz.) Jeswiet
Source: Sci Rep. 2019 Feb 21;9:2528. doi: 10.1038/s41598-019-38710-8 (PMC6385618; doi:10.1038/s41598-019-38710-8)
Supplement: Supplementary file 1 — Dataset 1 [file 41598_2019_38710_MOESM1_ESM.pdf]

Chromosome transmission in BC_4 progenies of intergeneric hybrids between *Saccharum* spp. and *Erianthus arundinaceus* (Retz.) Jeswiet

Shan Yang[1,2], Kai Zeng[1], Ke Chen[1], Jiayun Wu[3], Qinnan Wang[3], Xueting Li[2], Zuhu Deng[1,2,4]*, Yongji Huang[1], Fei Huang[1], Rukai Chen[2] & Muqing Zhang[4]

[1]National Engineering Research Center for Sugarcane, Fujian Agriculture and Forestry University, Fuzhou, China, 350002

[2]Key Lab of Sugarcane Biology and Genetic Breeding, Ministry of Agriculture, Fujian Agriculture and Forestry University, Fuzhou, China, 350002

[3]Guangdong Provincial Bioengineering Institute, Guangzhou Sugarcane Industry Research Institute, Guangzhou, China, 510316

[4]State Key Laboratory for protection and utilization of subtropical agro-bioresources, Guangxi University, Nanning, China, 530004

***Correspondence**
Zuhu Deng
dengzuhu@163.com

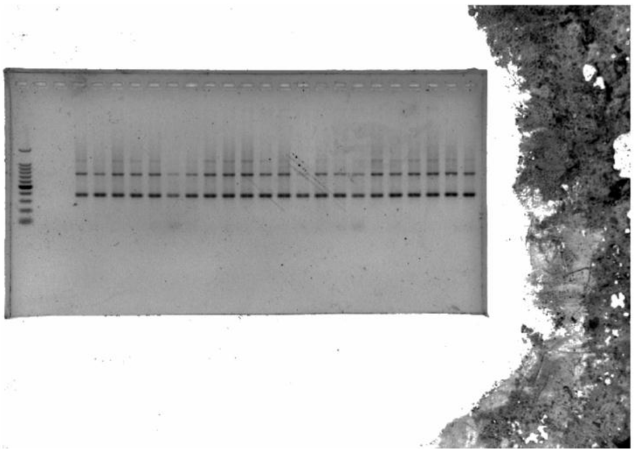

Figure 2_1

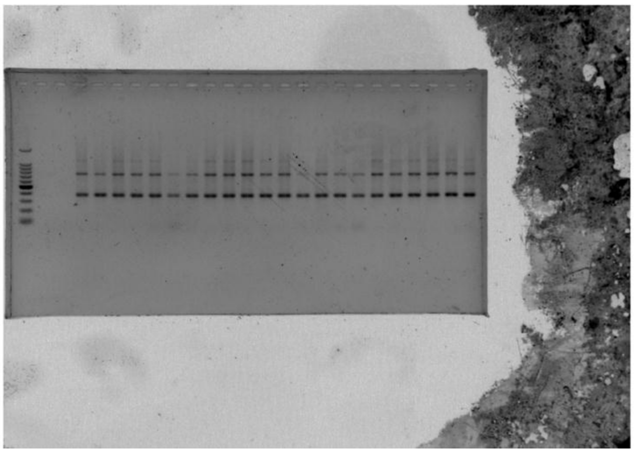

Figure 2_1_1

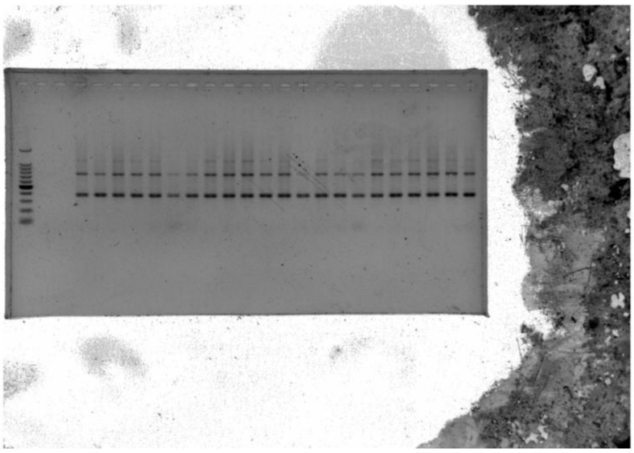

Figure 2_1_2

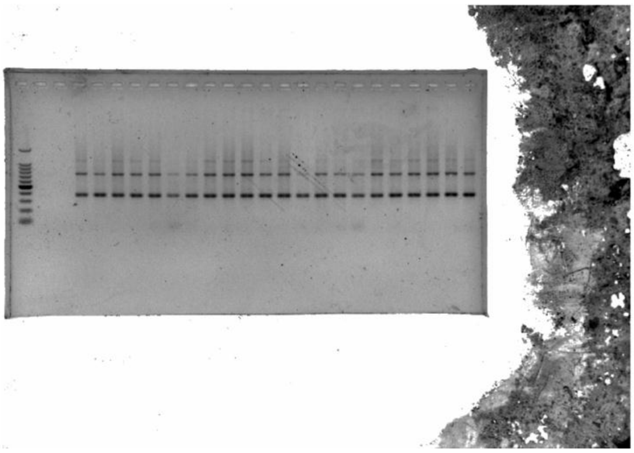

Figure 2_1_3

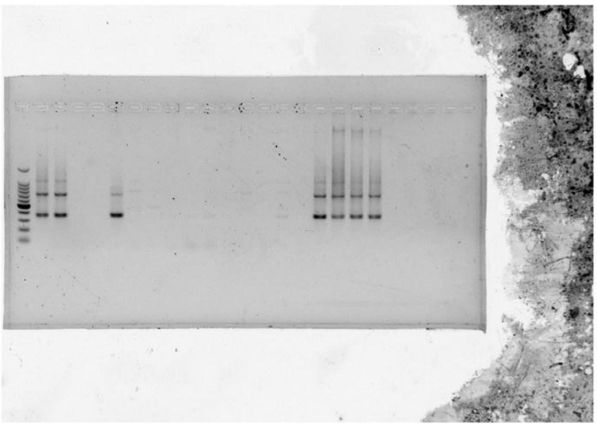

Figure 2_2

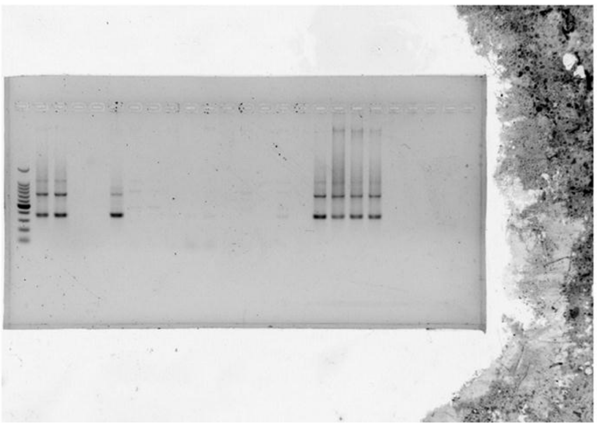

Figure 2_2_1

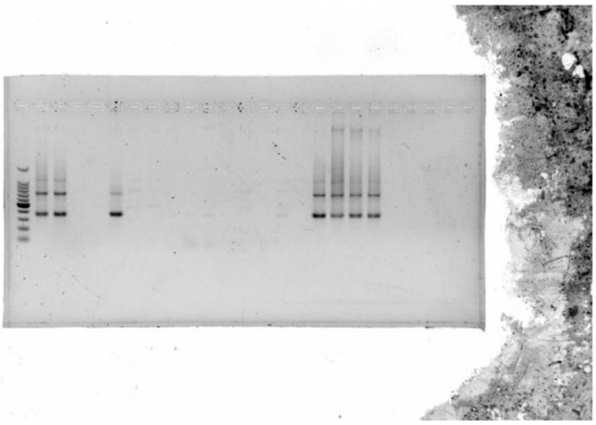

Figure 2_2_2

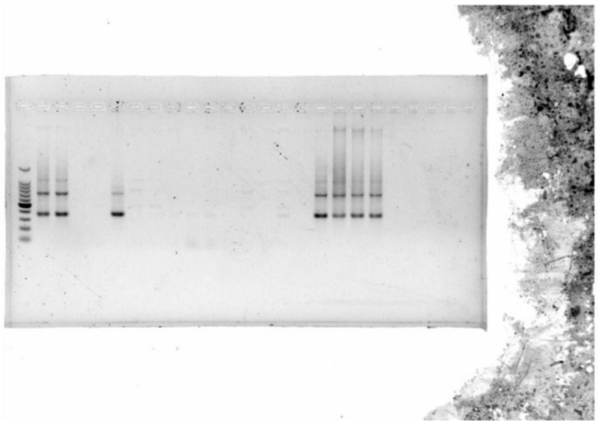

Figure 2_2_3

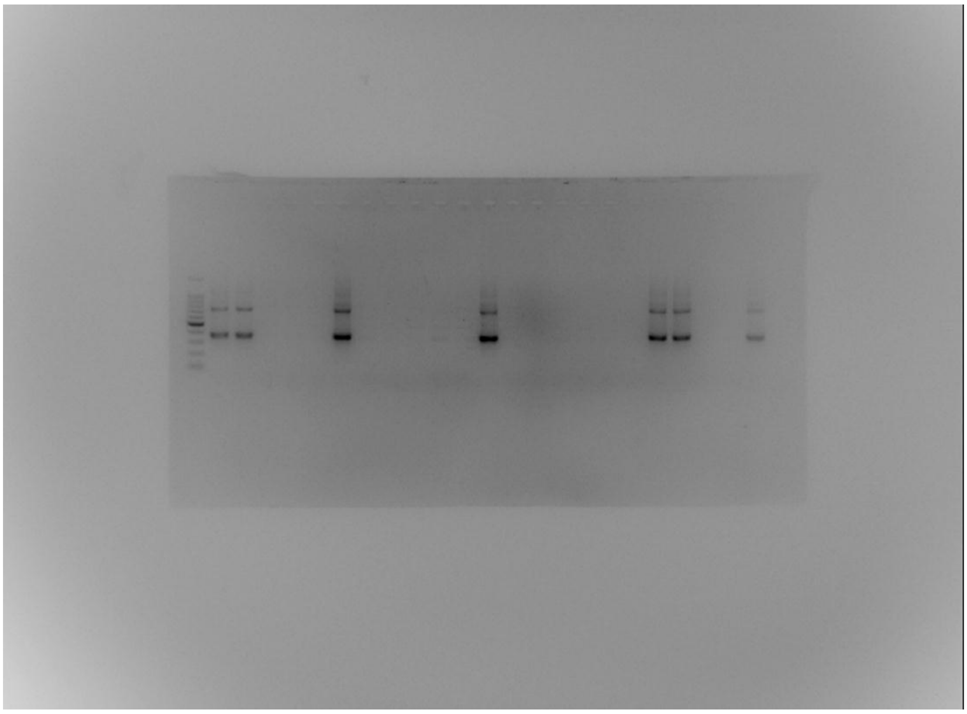

Figure 3_1

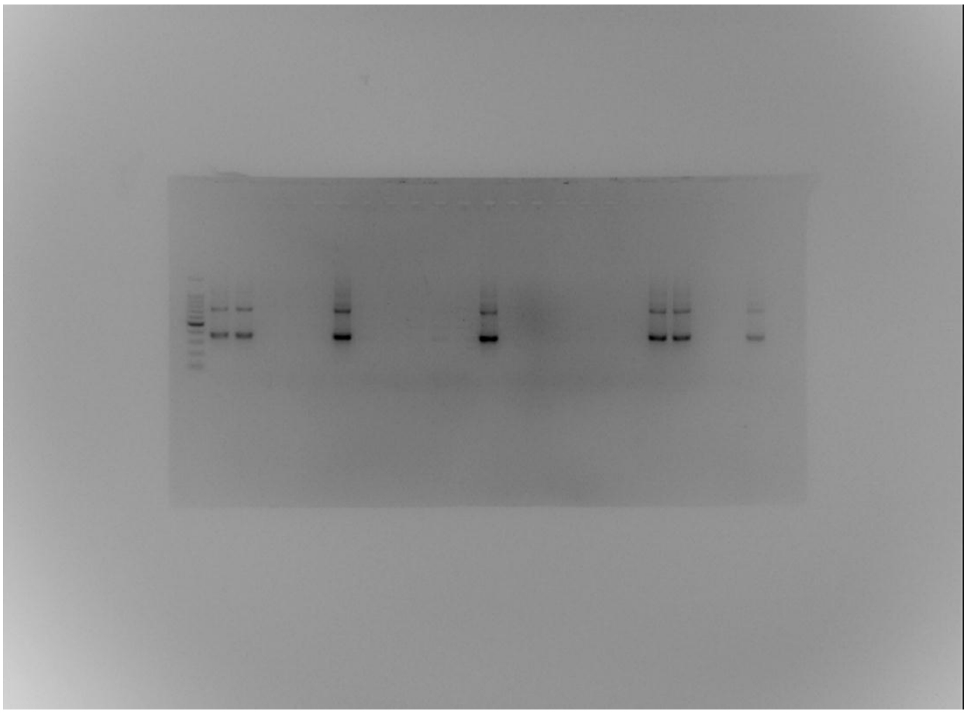

Figure 3_1_1

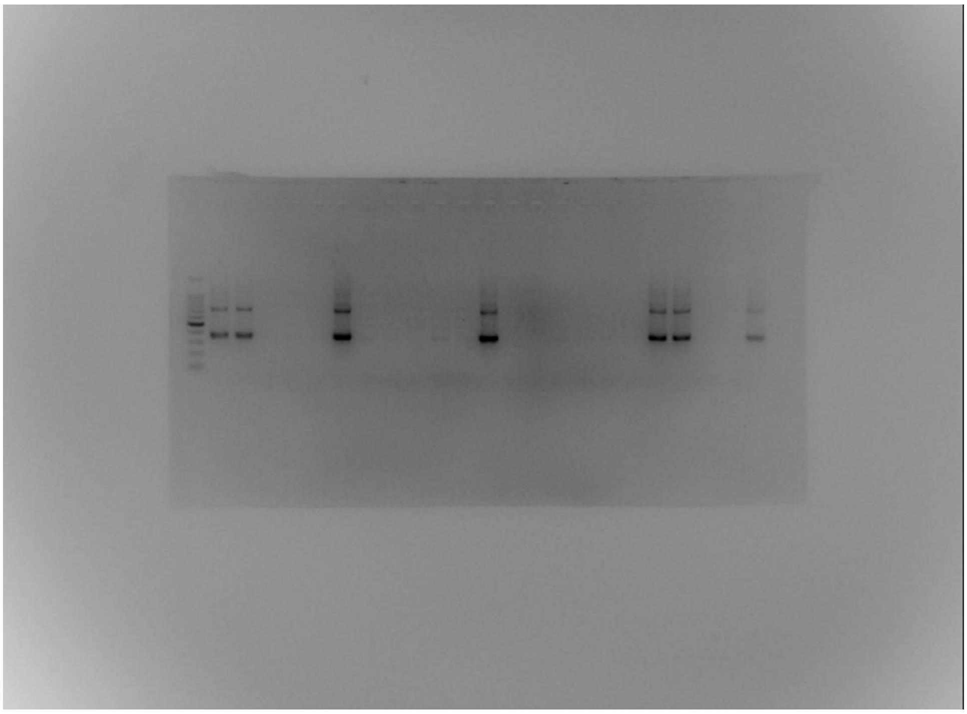

Figure 3_1_2

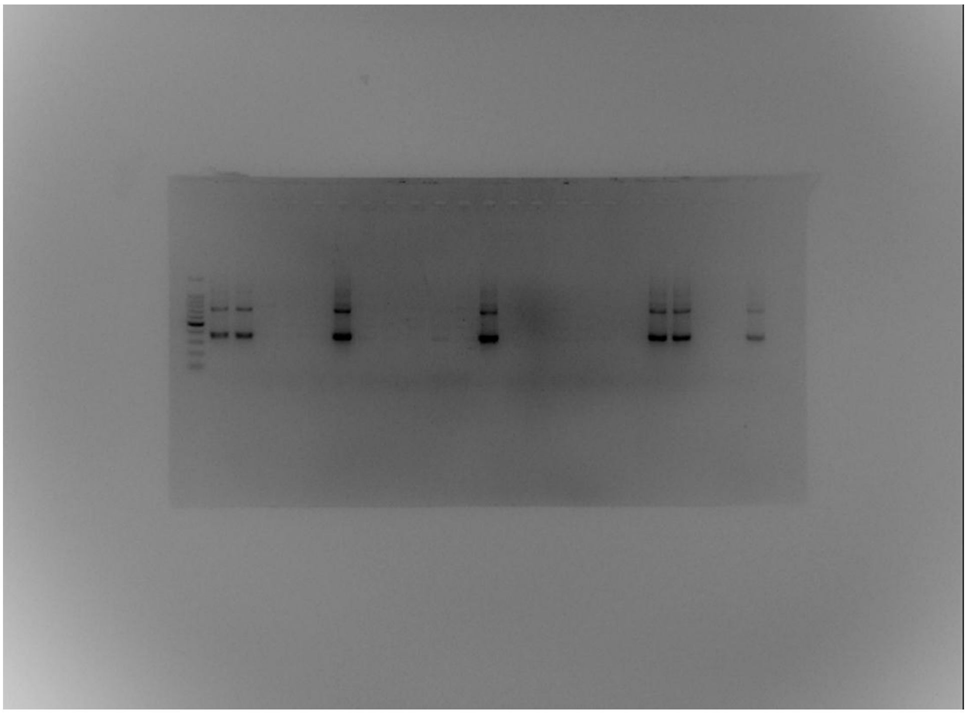

Figure 3_1_3

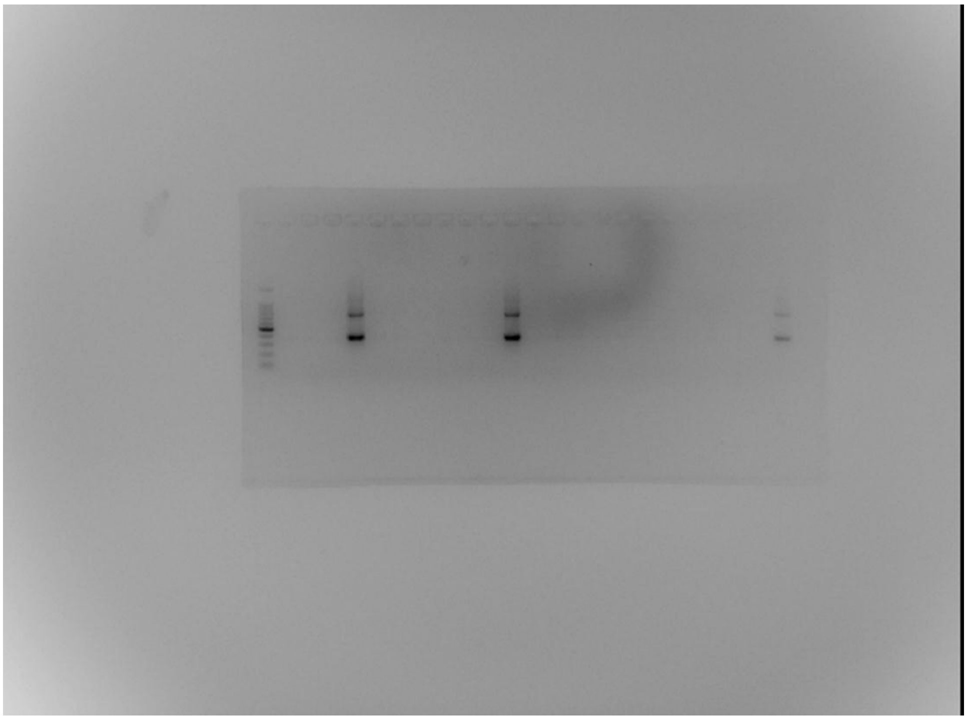

Figure 3_2

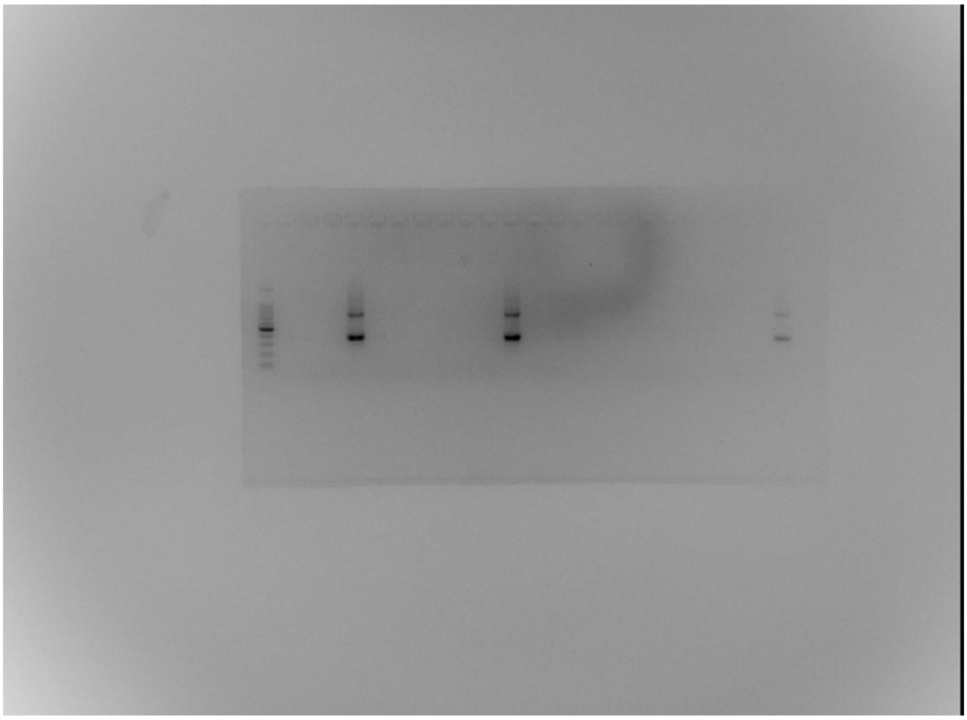

Figure 3_2_1

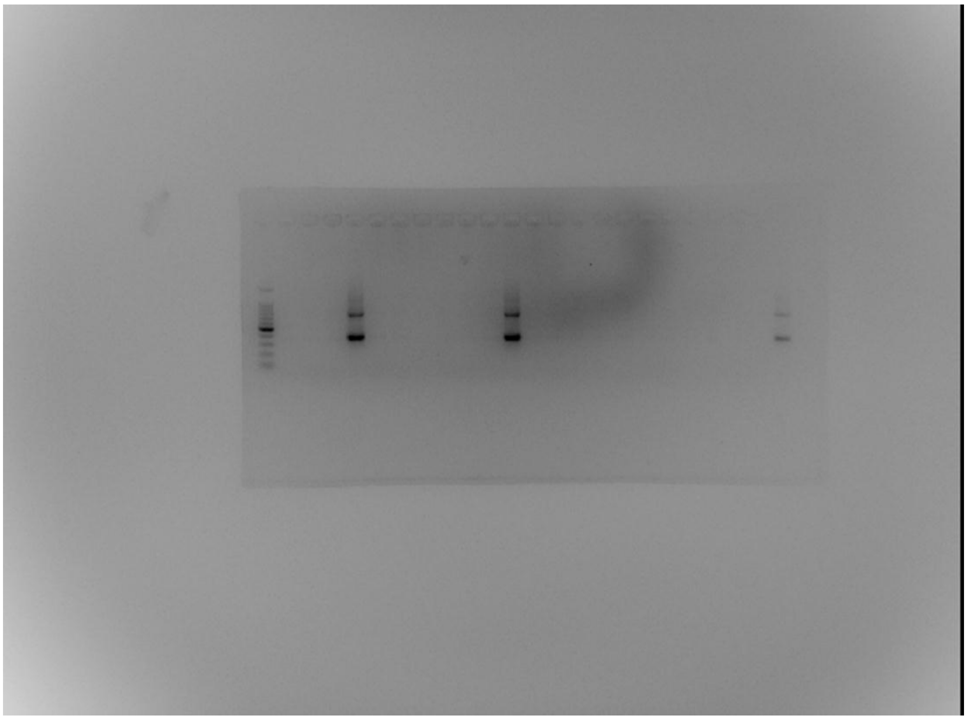

Figure 3_2_2

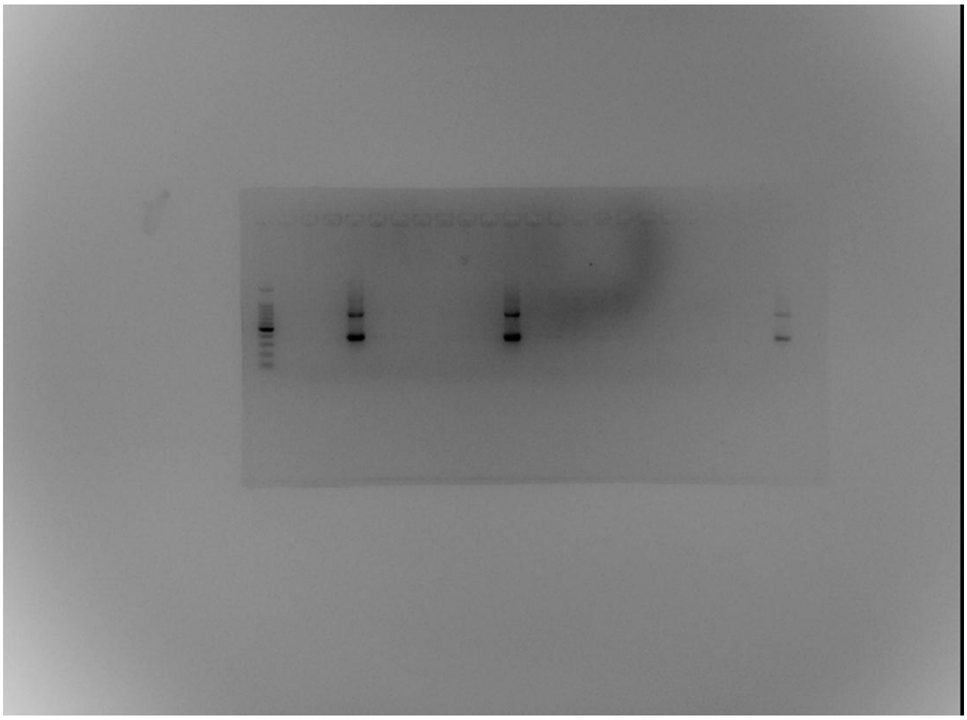

Figure 3_2_3
