# Supplementary material for: Chromosome transmission in BC4 progenies of intergeneric hybrids between Saccharum spp. and Erianthus arundinaceus (Retz.) Jeswiet
Source: Sci Rep. 2019 Feb 21;9:2528. doi: 10.1038/s41598-019-38710-8 (PMC6385618; doi:10.1038/s41598-019-38710-8)
Supplement: Supplementary file 2 — Dataset 2 [file 41598_2019_38710_MOESM2_ESM.pdf]

Chromosome transmission in BC_4 progenies of intergeneric hybrids between *Saccharum* spp. and *Erianthus arundinaceus* (Retz.) Jeswiet

Shan Yang[1,2], Kai Zeng[1], Ke Chen[1], Jiayun Wu[3], Qinnan Wang[3], Xueting Li[2], Zuhu Deng[1,2,4]*, Yongji Huang[1], Fei Huang[1], Rukai Chen[2] & Muqing Zhang[4]

[1]National Engineering Research Center for Sugarcane, Fujian Agriculture and Forestry University, Fuzhou, China, 350002

[2]Key Lab of Sugarcane Biology and Genetic Breeding, Ministry of Agriculture, Fujian Agriculture and Forestry University, Fuzhou, China, 350002

[3]Guangdong Provincial Bioengineering Institute, Guangzhou Sugarcane Industry Research Institute, Guangzhou, China, 510316

[4]State Key Laboratory for protection and utilization of subtropical agro-bioresources, Guangxi University, Nanning, China, 530004

***Correspondence**
Zuhu Deng
dengzuhu@163.com

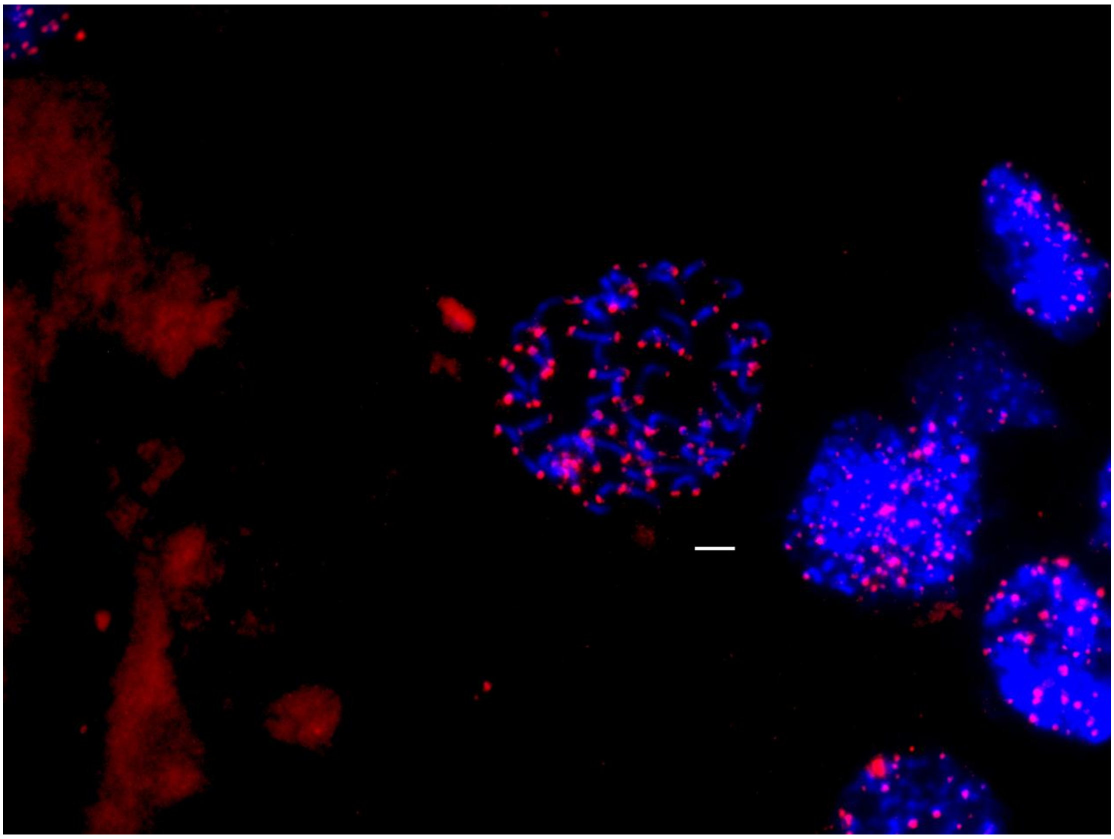

Figure 1A

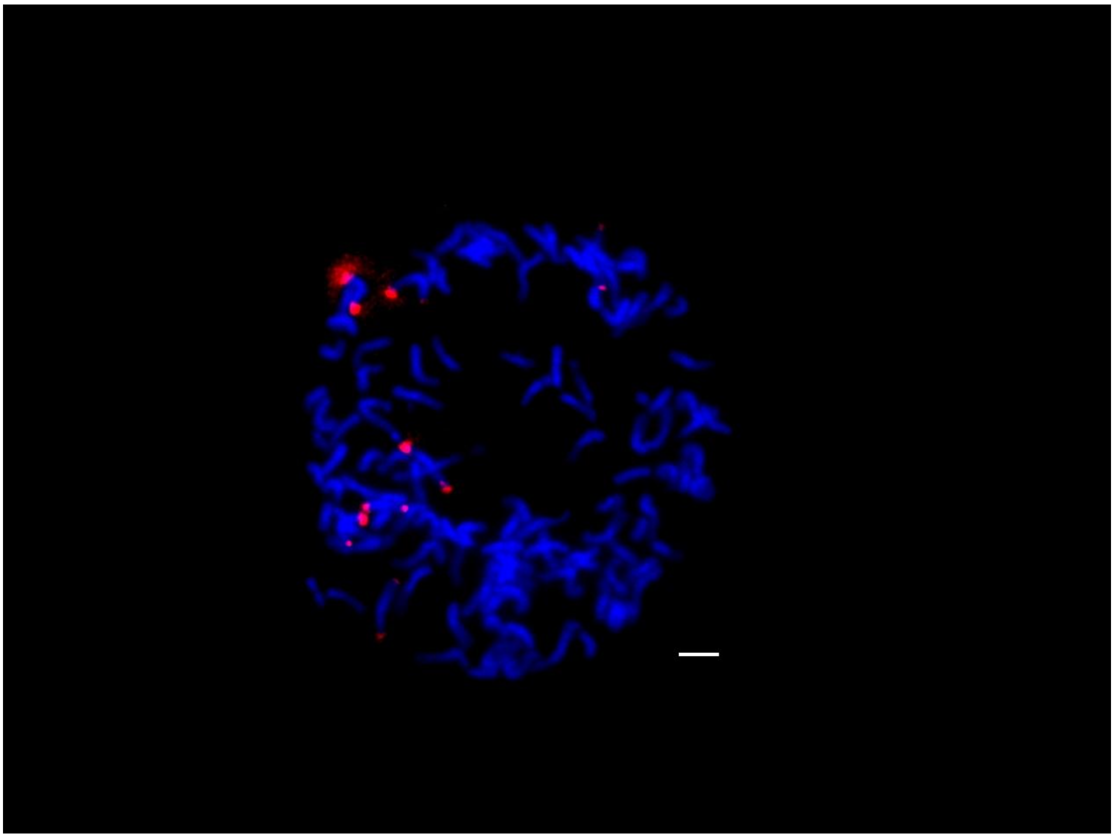

Figure 1B

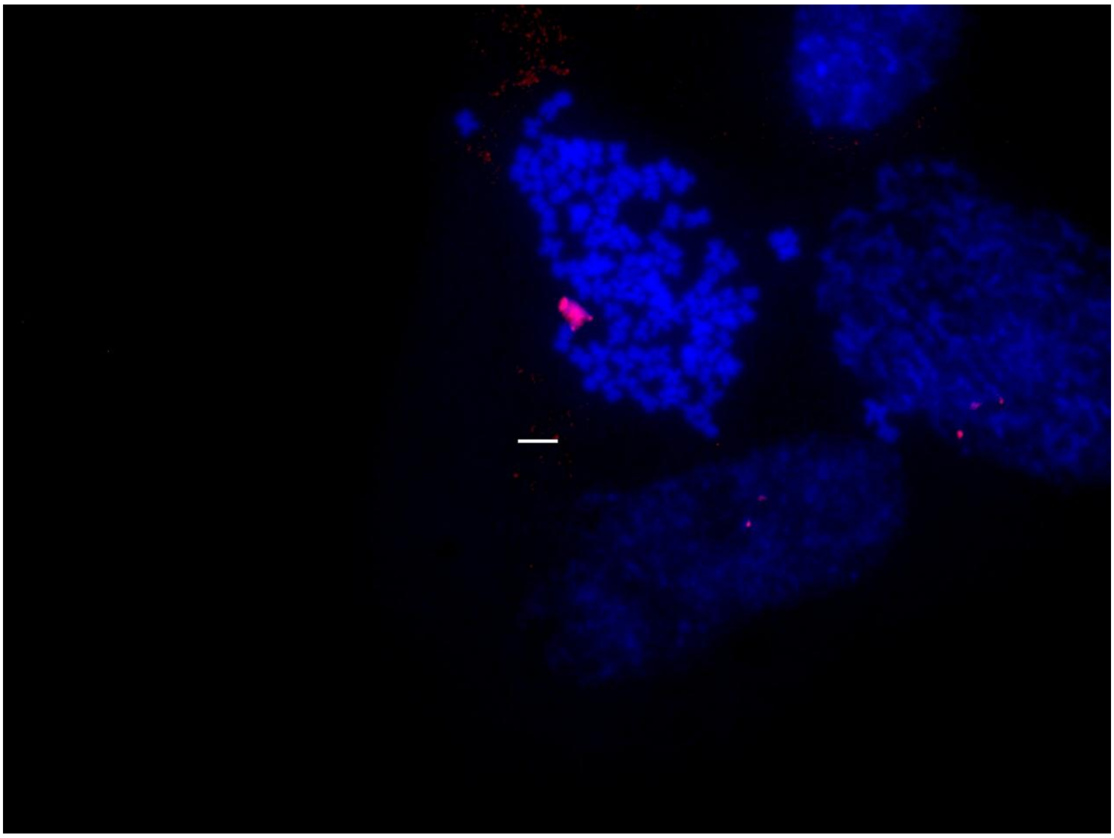

Figure 4A

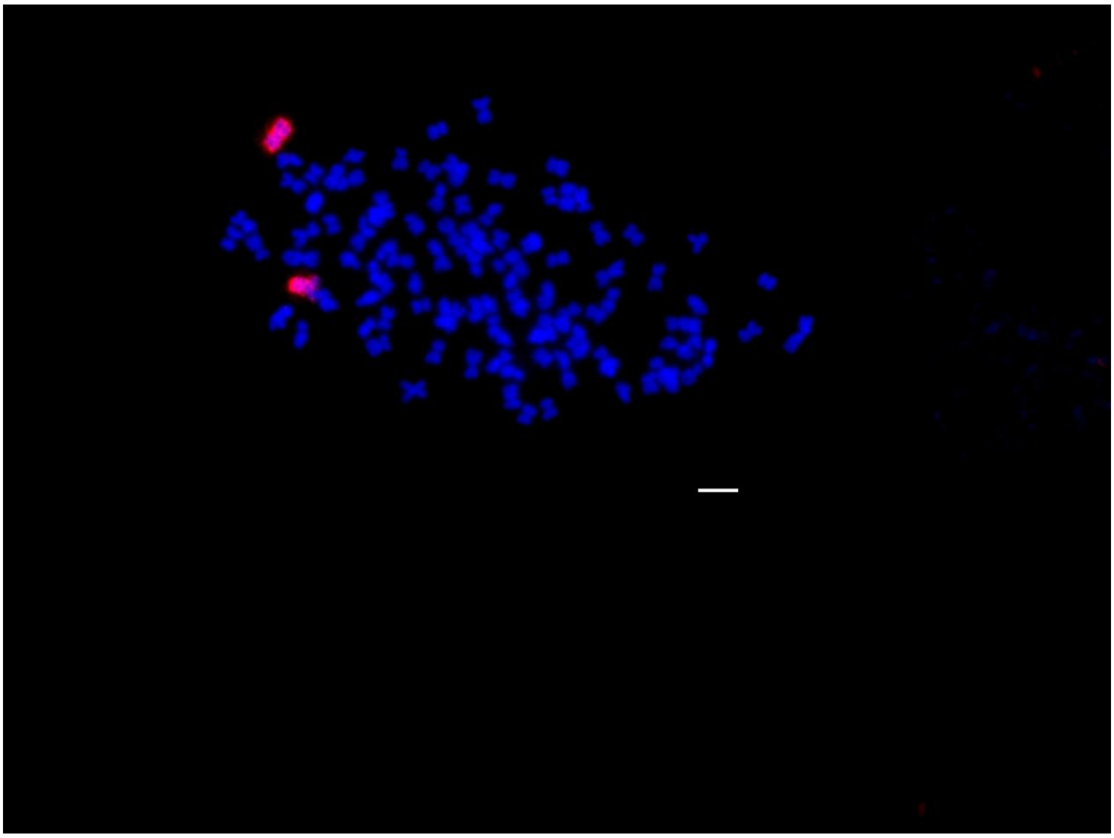

Figure 4B

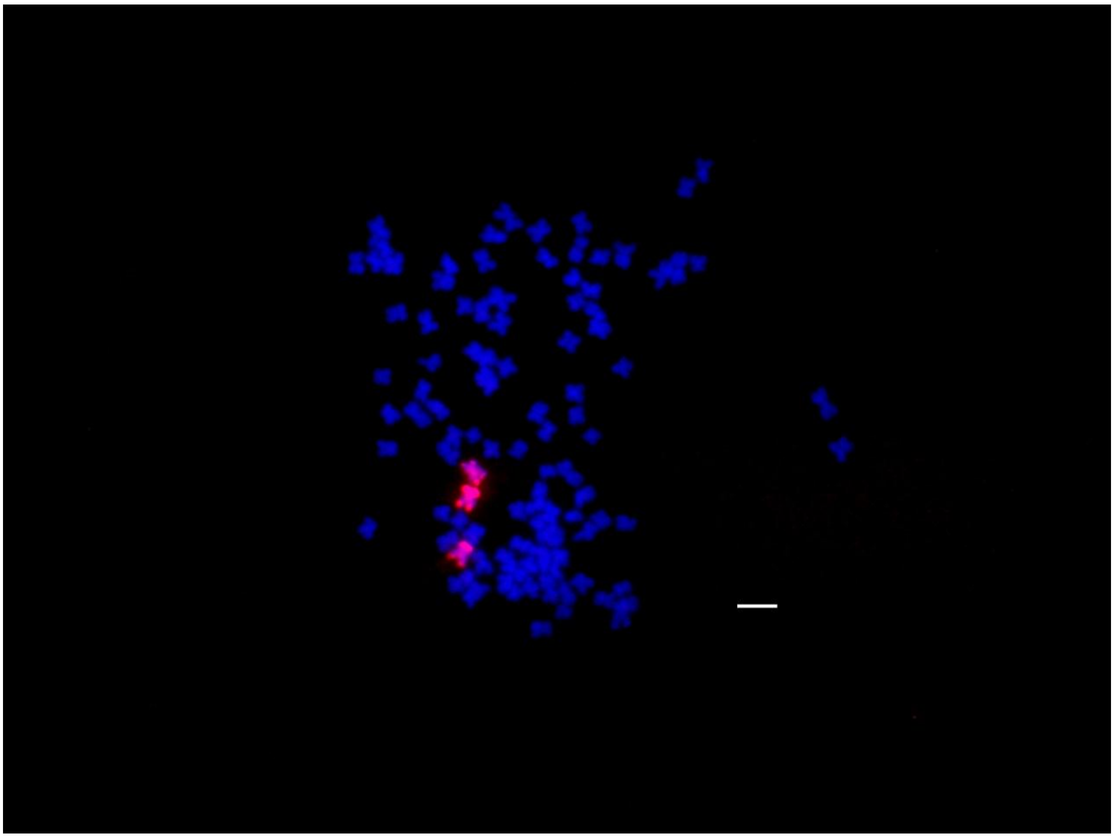

Figure 4C

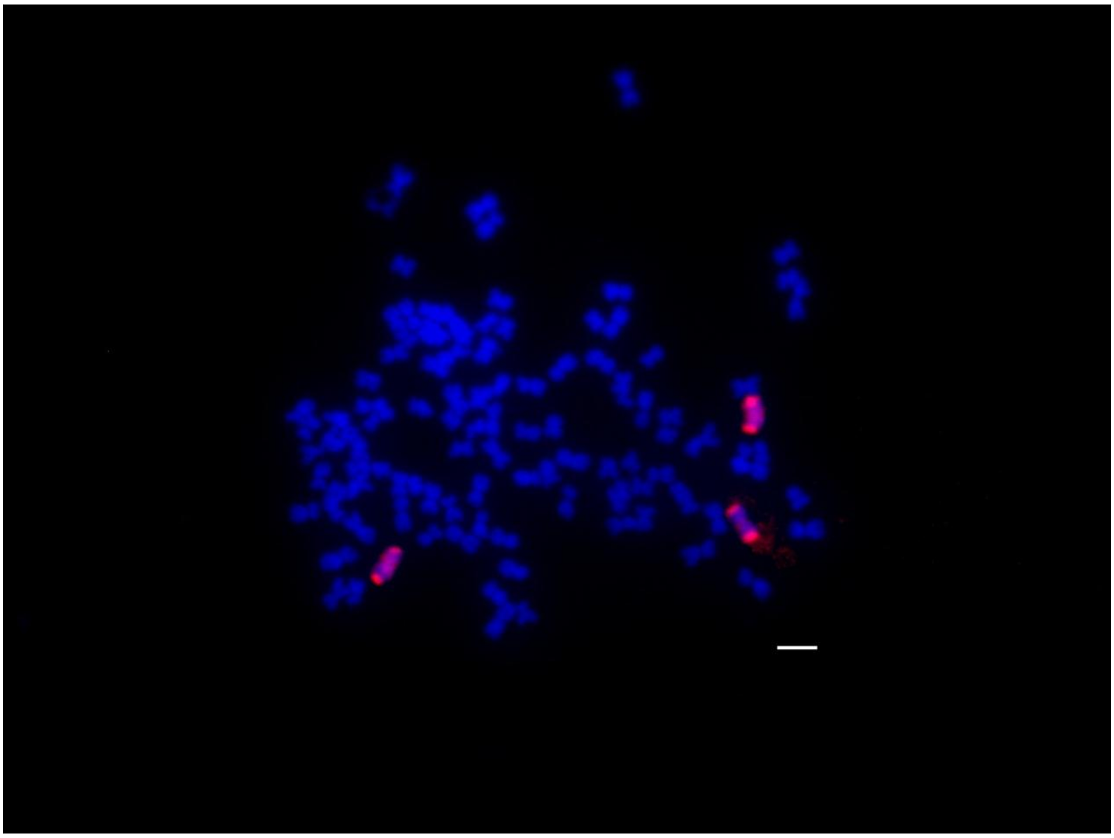

Figure 4D

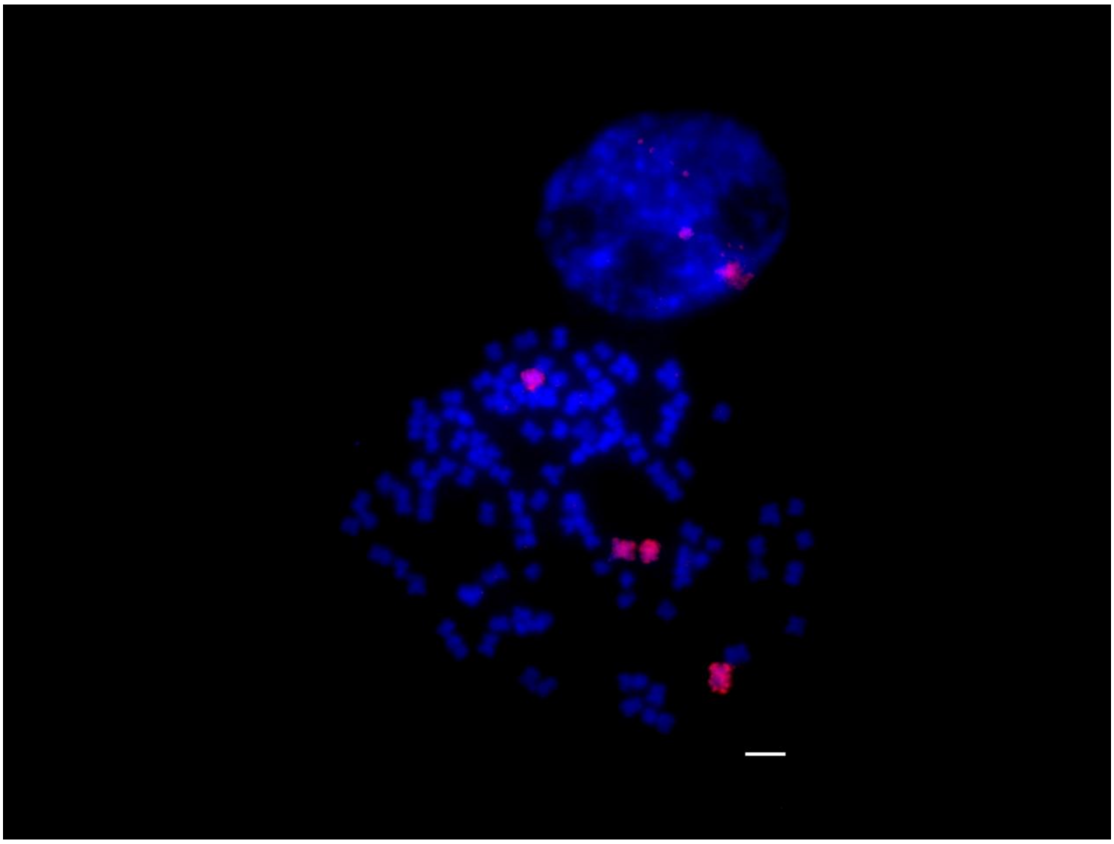

Figure 4E

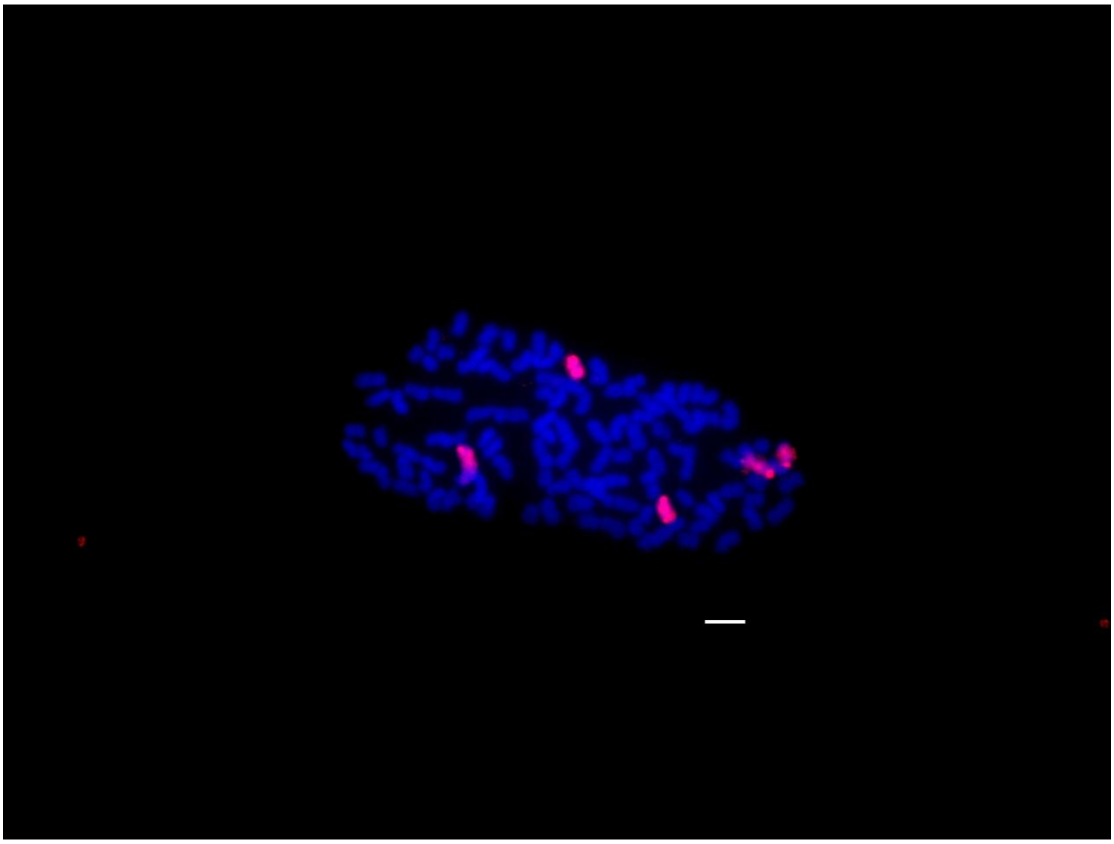

Figure 4F

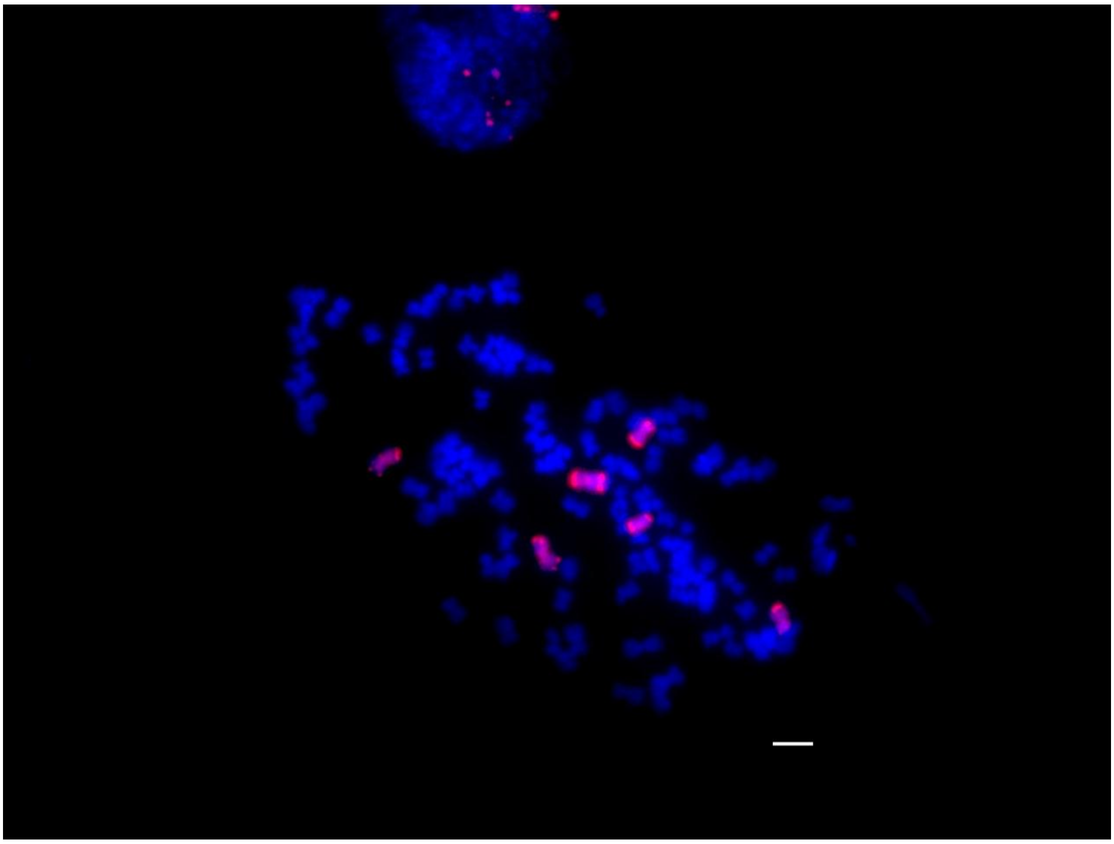

Figure 4G

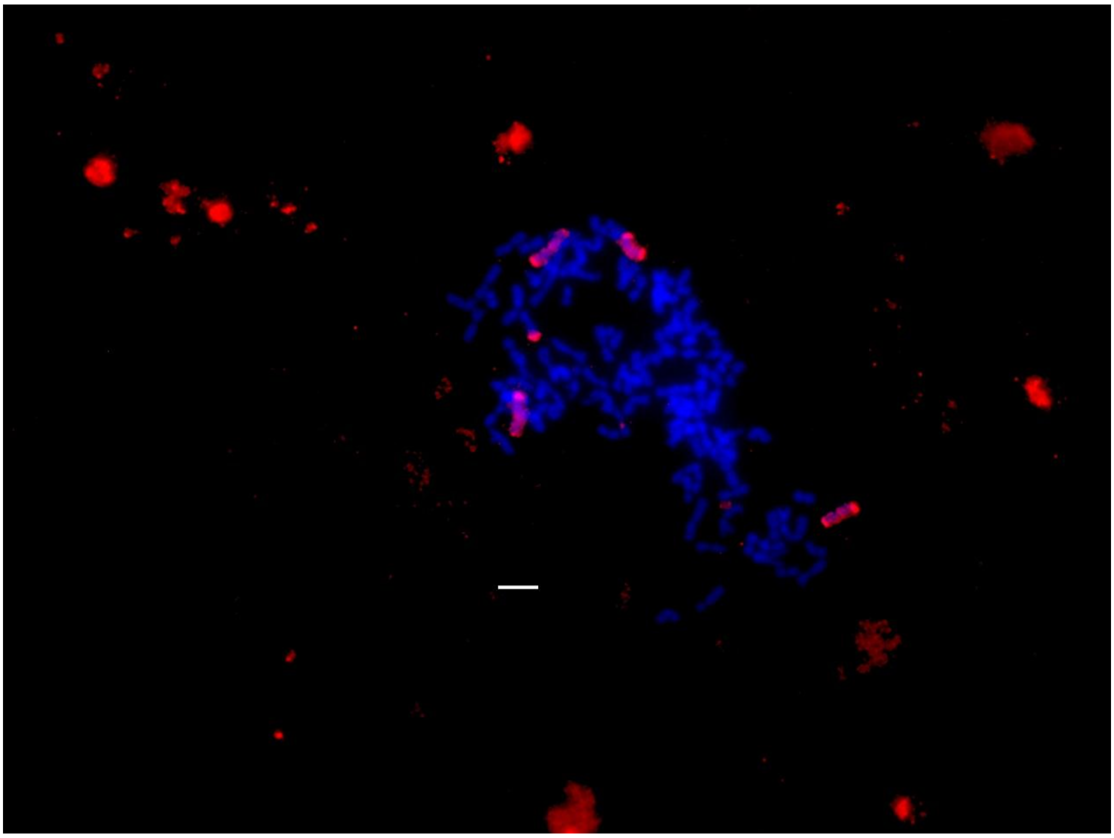

Figure 4H

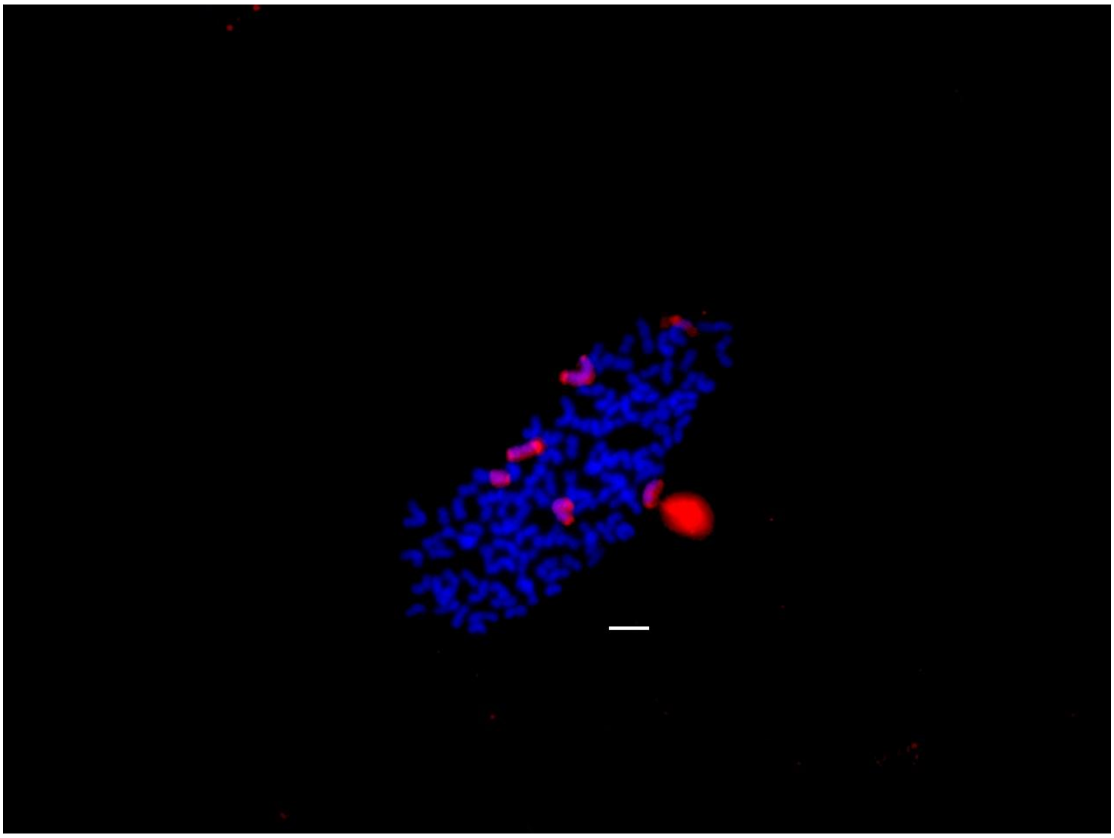

Figure 4I

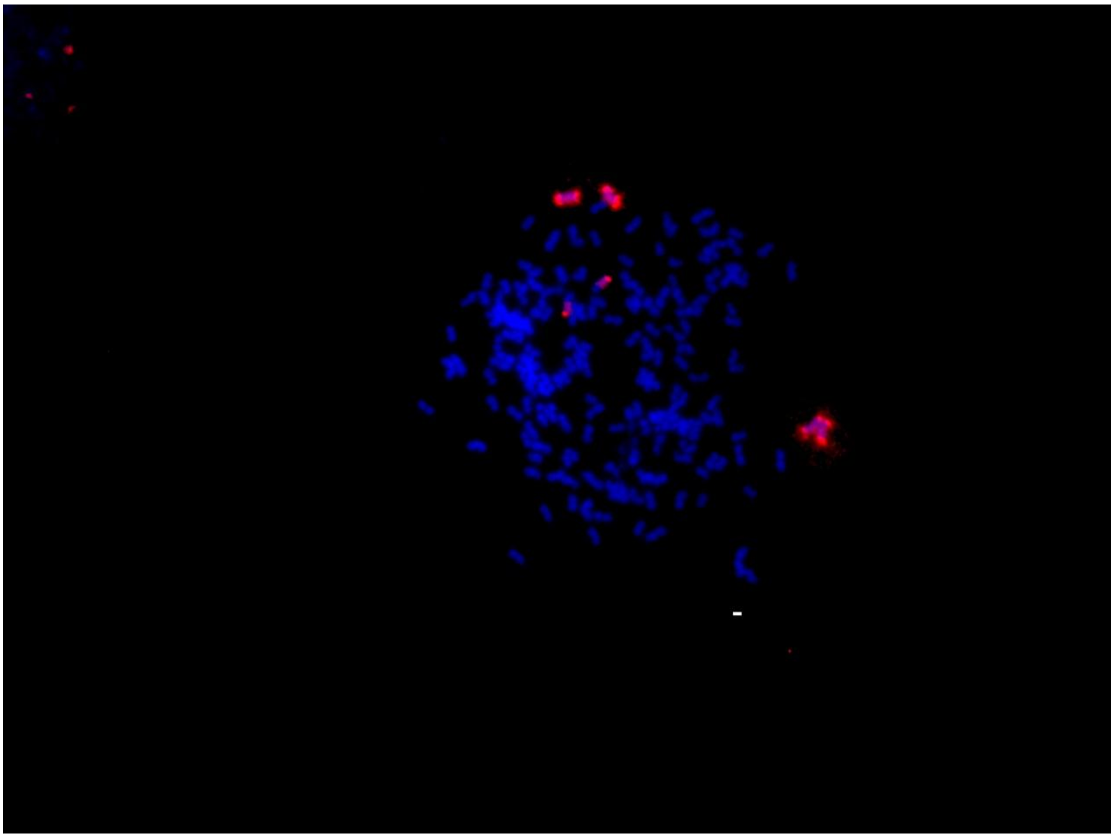

Figure 4J
